# Prevalence of work-related physical and psychosocial risks factors: a cross-sectional study of social welfare workers in Brazil

**DOI:** 10.47626/1679-4435-2023-1150

**Published:** 2024-11-14

**Authors:** Mariana Ferreira Justino, Viviane Gontijo Augusto, Iranise Moro Pereira Jorge, Lígia Prado Maríngolo, Lívia Maria Roncoleta, Fabiana Caetano Martins Silva e Dutra

**Affiliations:** 1 Center for Studies and Research on Work, Social Participation, and Health, Universidade Federal do Triângulo Mineiro (UFTM), Uberaba, MG, Brazil; 2 Physical Therapy Program, Universidade do Estado de Minas Gerais, Divinópolis, MG, Brazil; 3 Department of Occupational Therapy, Universidade Federal do Paraná, Curitiba, PR, Brazil; 4 Department of Occupational Therapy, Instituto de Ciências da Saúde, UFTM, Uberaba, MG, Brazil

**Keywords:** occupational health, workplace, diagnostic self-evaluation, workplace violence, social work, work, saúde do trabalhador, local de trabalho, autoavaliação diagnóstica, violência no trabalho, serviço social, trabalho

## Abstract

**Introduction:**

In Brazil, social service employees are exposed to poor work conditions, mental strain, and there is a lack of workforce, which contributes for the accumulation of tasks and to work overload.

**Objectives:**

To describe the prevalence of socioeconomic and occupational characteristics, working conditions and self-reported health of social service employees; and verify the relationship between working conditions and the workers self-reported health.

**Methods:**

The cross-sectional study evaluated sociodemographic and occupational characteristics, lifestyle and health information of social service employees; and used the Working Environment Assessment Protocol to assess their working conditions. The data was analyzed descriptively and by the chi-square test.

**Results:**

A total of 41 employees participated in this study, which corresponds to 60.3% of the total number of social service employees in the city. The majority was female (65.9%), aged 40.72 (standard deviation = 14.83), more than 8 years of schooling (82.9%), and occupying higher level functions (psychologists and social workers). Regarding health characteristics, 56.1% of participants practiced physical activities; 70.7% evaluated their own health as good or very good; and 43.9% related musculoskeletal pain. Not having a dining room; and temperature, ventilation, equipment, material resources and furniture were the most reported working conditions as inadequate. There was also an association between episodes of aggression and insecurity with self-perception of health.

**Conclusions:**

The results suggest that there could be a relationship between precarious working conditions and health. Specifically, this study indicated an association between poor working conditions and negative self-perception of health, insecurity and episodes of violence at work.

## Abstract (pt)

**Introdução:**

No Brasil, os trabalhadores do serviço social estão expostos a más condições de trabalho, desgaste mental e falta de mão de obra, o que contribui para o acúmulo de tarefas e para a sobrecarga de trabalho.

**Objetivos:**

Descrever a prevalência de características socioeconômicas e ocupacionais, as condições de trabalho e a percepção de saúde autorreferida entre trabalhadores do serviço social e verificar a relação entre as condições de trabalho e a saúde autorreferida dos trabalhadores.

**Métodos:**

Este estudo transversal avaliou características sociodemográficas e ocupacionais e informações sobre o estilo de vida e de saúde dos trabalhadores de serviços sociais, usando o Protocolo de Avaliação do Ambiente de Trabalho para avaliar as condições de trabalho. Os dados foram analisados descritivamente e pelo teste do qui-quadrado.

**Resultados:**

Participaram deste estudo 41 servidores, o que corresponde a 60,3% do total de servidores de serviço social do município. A maioria era do sexo feminino (65,9%), com idade de 40,72 anos (desvio-padrão = 14,83), com mais de 8 anos de estudo (82,9%) e ocupando cargos de nível superior (psicólogos e assistentes sociais). Em relação às características de saúde, 56,1% dos participantes praticavam atividades físicas; 70,7% avaliaram a própria saúde como boa ou muito boa; e 43,9% relataram dor musculoesquelética. A falta de um refeitório, a temperatura, a ventilação, os equipamentos, os recursos materiais e os móveis foram as condições de trabalho mais relatadas como inadequadas. Também houve associação entre episódios de agressão e insegurança com a autopercepção de saúde.

**Conclusões:**

Os resultados sugerem que há relação entre condições precárias de trabalho e saúde. Este estudo indicou associação entre más condições de trabalho e autopercepção negativa de saúde, insegurança e episódios de violência no trabalho.

## INTRODUCTION

Work and its organization have undergone significant changes in recent years, resulting in changes in the way people work, the profile of occupational diseases, and the prevalence of indicators such as sickness rates, disability and early retirement.^1^ Recent global events, in particular the COVID-19 pandemic, have exacerbated existing psychosocial risks and introduced new ones, highlighting the need to protect workers’ health more comprehensively.^2^ Job demands include several physical and ergonomic risk factors, such as excessive posture, lack of breaks, and prolonged periods in the same position.^3^ In addition, working conditions include psychosocial factors such as task control, autonomy and decision-making, psychological demands, social support, organizational climate and workplace violence.^4^ Both physical and psychosocial demands influence the work environment, either positively or negatively, and affect workers’ health. These developments have spurred national and international debates on the need to prioritize physical, ergonomic and psychosocial risks in workplace policies, strategies, and interventions.^2^

### BACKGROUND

Specific groups of workers with high physical and psychosocial demands face greater risks and vulnerabilities, leading to high rates of morbidity and mortality, absenteeism, and disability retirement.^3-5^ In Brazil, health workers are particularly affected because their work is often associated with poor working conditions, decision-making pressure, lack of support, and excessive workloads, all of which contribute to physical and emotional exhaustion.^6^ In addition to psychosocial factors, inadequate working conditions - such as poor infrastructure, inadequate furniture, lack of proper equipment, and unhealthy working environments - further impede job performance and negatively affect workers’ health.^7^ Situations of workplace violence, both physical and verbal, can trigger feelings of anger, disappointment, fear, and distress. These negative experiences can interfere with job functioning and lead to intentions to leave the job or change careers.^8^

This reality is also true for workers in the Brazilian Sistema Único de Assistência Social (SUAS). SUAS, through the Centro de Referência de Assistência Social (CRAS), aims to reduce social inequalities by supporting vulnerable populations.^9^ Each CRAS has the capacity to offer assistance to 500 families in vulnerable situations and includes a multidisciplinary health team consisting of at least a coordinator, a social welfare worker, a psychologist, and technicians with a high school education.^10^

Among the activities developed by CRAS are: guiding and improving community, social, and family interactions; mapping individuals in situations of risk; referring individuals and families to social assistance and public policy services.^10^ CRAS can identify various problems such as poverty, threats and abandonment, domestic violence, drug abuse, crime, low socioeducational levels, and loss of rights.^10^ CRAS workers are also exposed to poor working conditions, psychological stress, and understaffing, which contribute to high workloads and make it difficult to perform effectively.^10,11^

### PURPOSE

Sick leave represents a significant financial burden in terms of lost productivity, the cost of hiring and training new workers, social security and welfare expenses, and additional health care costs.^5^ As a workplace health indicator, monitoring working conditions can reveal exposure to physical and psychosocial risks in different sectors. These indicators can help identify prevalent and common health problems, predict disability, and guide targeted investment in social welfare workers. Although there is an extensive literature on working conditions and health, specific research focusing on social service workers is limited. Identifying the job characteristics associated with worker illness could be critical for implementing interventions to reduce sickness-related costs among social welfare workers and promote safer, healthier, more functional working conditions.

In this context, this study aims to: (i) describe the socioeconomic and occupational characteristics, working conditions, and self-reported health of Brazilian CRAS social welfare workers, and (ii) assess the relationship between working conditions and social welfare workers self-reported health.

## METHODS

The present observational, cross-sectional study focuses on social workers in a large city that serves the socially vulnerable population of 27 cities in southeastern Brazil. The city has eight CRAS units, each equipped with a reception area, one or more assessment rooms, a room for activities with groups and/or families, an administrative room, a dining room, and bathrooms.

A total of 65 social welfare workers were employed as professionals in the CRAS units. Inclusion criteria were workers of both sexes, 18 years of age or older, and active for at least one year in any position or function at CRAS. Retired social workers and social workers on leave were excluded. Data were collected from November 2019 to January 2020, during visits by researchers to all eight CRAS units. Participants were provided with information about the aims and methods of the study and were invited to participate voluntarily. Participants signed an informed consent form and completed the survey at their respective CRAS units, depending on their availability.

Of the 65 social welfare workers, 41 (63.07%) agreed to participate in the study through convenience sampling. This sampling technique was chosen due to the availability of active social welfare workers at the workplace during the study period. To calculate the power of this sample, a significance level of α = 0.05, a large conventional effect size (ω = 0.35), a sample size (n) of 40, and a degree of freedom (df) of 1 were considered. Using G*Power® 3.1.9.7 software, the calculated power of the sample (n = 40) was 0.9125.

Data collection consisted of three questionnaires: (1) a sociodemographic, occupational, life habit questionnaire adapted from Roncoleta et al.,^12^ which collects information on sociodemographic aspects (sex, age, education level, marital status, income), lifestyle habits (physical activity, smoking, amount of sleep), and occupational data (position, time in position, shifts, working hours); (2) the Health Perception Questionnaire (HPQ); and (3) the Work Environment Evaluation Protocol.

Health perceptions were assessed by a series of questions adapted from Barbosa et al.,^13^ including aspects such as musculoskeletal pain (duration, intensity, and location), perception of tiredness, self-rating of current health status as positive or negative, involvement and satisfaction with daily life activities (DLAs), and a list of physical and mental health problems experienced in the past 30 days.

The Work Environment Evaluation Protocol was developed by Barbosa et al.^13^ In the present study, items from this protocol specific to the CRAS context were used, such as work environment conditions (ventilation, temperature, lighting, furniture, equipment, noise, dining room/canteen, restroom, personal effects lockers, and material resources), physical demands (posture that leads to pain or discomfort, standing for long periods of time, sitting for long periods of time, walking too much, carrying or lifting weight, working without breaks, rotating shifts, night shifts), and psychosocial aspects (little to no time to eat, employer does not provide food, safety, and aggression episodes). Protocol consisted of 25 questions with a 5-point Likert scale (1 = never; 2 = rarely; 3 = infrequently; 4 = frequently; and 5 = very frequently). For this study, the response options were converted into two categories: adequate (if responses were frequently or very frequently) and inadequate (if responses were never, rarely, or infrequently).

All data were analyzed descriptively. Data were first adjusted for missing values and outliers. Less than 5% of data were missing, and the multiple imputation method was used to account for these missing data.^14^ The reliability of each item of HPQ and Work Environment Evaluation Protocol was assessed by using the kappa coefficient with quadratic weighting (kw). Interpretation of kappa coefficient of agreement followed the criteria proposed by Landis & Koch^15^: almost perfect (0.81 to 1.00), strong (0.61 to 0.80), moderate (0.41 to 0.60), regular (0.21 to 0.40), discrete (0 to 0.20), and poor (< 0). Association between working conditions and health perceptions was tested by using the chi-square test. Statistical analyses were performed by using IBM^®^ Statistical Package for the Social Sciences (SPSS) software, version 20.0, with a significance level of α = 0.05 for all analyses.

The present research was approved by the Research Ethics Committee at Universidade Federal do Triângulo Mineiro (under CAAE no. 52647216.7.0000.5154 and approval no. 1,774,870) and all participants signed a free and informed consent form. As an ethical guarantee, participant information is kept confidential. In order to ensure the security and confidentiality of the information provided, all instrument protocols were archived under the responsibility of the research group conducting the study. Participants received no compensation and participation in the study was completely voluntary. The study followed the Strengthening the Reporting of Observational Studies in Epidemiology (STROBE) guidelines for reporting observational data.^16^ To reduce potential information bias, raters were pretrained in the use of the instruments, and participants were blinded to the aims of the study data analysis.

## RESULTS

### CHARACTERISTICS OF THE PARTICIPANTS

Most participants were women (65.9%), with 8 or more years of formal education (82.9%). Almost half were married (41.5%) and most had children (51.2%). The mean age was 40.72 years (standard deviation [SD] = 14.83), and the mean monthly income was R$ 2,071.03 (SD = 1,152.42). Of the participants, 58.5% worked only one shift, with a mean daily working time of 6.9 hours (SD = 1.09), and 22% were psychologists or social welfare workers (Table 1).

**Table 1:** Sociodemographic, occupational, and health characteristics of the CRAS workers (n = 41)

Variables	Frequency (n)	Percentage (%)
Functions		
Social agent	5	12.2
Administrative assistant	3	7.3
Social worker	9	22.0
Canteen worker	1	2.4
Manager	5	12.2
Arts instructor	1	2.4
Psychologist	9	22.0
Receptionist	1	2.4
Janitors	5	12.2
CRAS		
I	6	14.6
II	3	7.3
III	10	24.4
IV	2	4.9
V	9	22.0
VI	1	2.4
VII	6	14.6
VIII	4	9.8
Sex		
Female	27	65.9
Male	14	34.1
Educational level, years		
Up to 8 of study	7	17.1
More than 8 of study	34	82.9
Marital status		
Without partner	24	58.5
With a partner	17	41.5
Children		
Does not have children	20	48.8
Has children	21	51.2
Shifts		
1	24	58.5
2	14	34.1
Physical activity		
Does not practice	18	43.9
Practices	23	56.1
Smoking		
No	38	92.7
Yes	3	7.3
Health perception		
Positive (very good, good)	29	70.7
Negative (regular, bad, very bad)	12	29.3
Musculoskeletal pain		
No	20	48.8
Yes	18	43.9
Sleeps bad		
No	27	65.9
Yes	14	34.1
Health problem		
Poor digestion	8	19.5
No appetite	2	4.9
Trembling hands	8	19.5
Tires easily	16	39.0
Headache	9	22.0
Thoughts on ending their own lives	3	7.3
Difficulties in making decisions	12	29.3
Feeling nervous, tense, preoccupied	20	48.8
Loss of satisfaction in DLAs	11	26.8

CRAS = Centro de Referência de Assistência Social; DLAs = daily life activities.

### HEALTH CHARACTERISTICS

The kw of the reliability assessment for HPQ indicated perfect reliability (kw = 0.859). A total of 56.1% of participants were physically active for at least 60 minutes an average of three times a week. Only 7.3% of social welfare workers were smokers, and the average hours of sleep per night was 7.1 hours (SD = 1.09). A total of 70.7% of social welfare workers self-rated their health as positive; 43.9% reported musculoskeletal pain with a mean intensity of 6.1 (SD = 1.99), and 87.5% of these cases were chronic pain (lasting more than 3 months). The most common pain locations were back/spine and legs/feet. Nearly half of the social welfare workers (48.8%) felt nervous, tense, and preoccupied; 39% reported tiring easily; 29.3% had difficulties in making decisions; 26.8% reported a loss of satisfaction in their DLAs; 22% experienced headaches; and 7.3% had thoughts about ending their own lives (Table 1).

### WORK CONDITIONS CHARACTERISTICS

Reliability scores for the Work Environment Evaluation Protocol ranged from regular to strong, with kw = 0.859 for items related to work environment conditions, kw = 0.473 for physical demands, and kw = 0.748 for psychosocial aspects.

Most CRAS units (97.6%) do not have a dining room, 78% have inadequate temperature, and 75.6% have poor ventilation and work equipment. Besides, 65.9% of CRAS units lack material resources, 63.4% have inadequate furniture, and 58.5% do not have personal effects lockers (Table 2).

**Table 2:** Work environment conditions and demands in Centro de Referência de Assistência Social (CRAS) units (n = 41)

Variables	Frequency (n)	Percentage (%)
Poor conditions of		
Ventilation	31	76.6
Temperature	32	78.0
Lighting	20	48.8
Furniture	26	63.4
Equipment	31	75.6
Noise	9	22.0
No dining room/canteen	2	4.9
No room to rest	40	97.6
No personal effects lockers	24	58.5
Material resources	27	65.9
Demands		
Posture that leads to pain or discomfort	23	56.1
Standing for long periods of time	14	34.1
Sitting for long periods of time	27	65.9
Walking too much	14	34.1
Carrying or lifting weight	12	29.3
Working without break	21	51.2
Rotating shifts	19	46.3
Night shifts	11	28.8
Psychosocial aspects		
Little to no time to eat	5	12.2
The employer does not offer food	35	85.4
Personal safety under threat	28	68.3
Personal effects safety under threat	22	53.7
Episodes of aggression or threats made by the user	11	26.8
Episodes of aggression or threats made by the companion or by a relative of the user	8	19.5
Episode of aggression or threats from colleagues or superiors to the users	3	7.3
Episode of aggression or threats between colleagues and/or superiors	4	9.8

In terms of the physical demands of the job, 65.9% of social welfare workers spend long periods of time sitting and 56.1% work in positions that cause pain or discomfort. In addition, 51.2% work without breaks and nearly half (46.3%) work rotating shifts. Among social welfare workers, 34.1% either walk or sit for long periods without breaks or posture changes, 29.3% carry or lift weights during the workday, and 28.8% work night shifts (Table 2).

Regarding psychosocial aspects, most social welfare workers (85.4%) are not provided with food by their employers and 12% have little or no time to eat. In addition, 68.3% of participants felt their personal safety was threatened at work, and more than half (53.7%) felt the security of their belongings was threatened. In addition, 26.8% of social workers have experienced episodes of aggression or threats from members of the public or relatives/companions of health service users. Episodes of aggression or threats between co-workers and/or their supervisors (9.8%) and from supervisors or co-workers to users (7.3%) were also reported (Table 2).

### ASSOCIATIONS BETWEEN THE CONDITIONS OF THE WORK AND THE PERCEPTION OF HEALTH

Poor ventilation conditions were significantly associated with poor sleep (p = 0.008), easy tiredness (p = 0.003), feelings of nervousness, tension, and preoccupation (p = 0.006), and loss of satisfaction with DLAs (p = 0.027). The loss of satisfaction with DLAs was significantly associated with precarious temperature conditions (p = 0.041) and lack of material resources (p = 0.042). Poor lighting conditions (p = 0.001) and lack of equipment (p = 0.032) were associated with the symptom of easy tiredness. Lack of material resources was also significantly associated with difficulty making decisions (p = 0.025) (Table 3).

**Table 3:** Health aspects of CRAS social welfare workers associated with poor work environment conditions (n = 41)

	Poor work environment conditions (p-value)						
	Ventilation	Temperature	Lighting	Furniture	Equipment	Noise	Resources
Musculoskeletal pain	0.282	0.152	0.373	0.465	0.432	0.218	0.314
Sleeping badly	0.008^*^	0.333	0.136	0.395	0.535	0.572	0.428
Poor digestion	0.083	0.573	0.623	0.627	0.642	0.644	0.257
No appetite	0.567	0.605	0.232	0.396	0.567	0.904	0.428
Trembling hands	0.642	0.427	0.623	0.627	0.358	0.644	0.435
Tires easily	0.003^*^	0.006^*^	0.001^*^	0.057	0.032^*^	0.146	0.091
Headache	0.058	0.080	0.202	0.572	0.283	0.395	0.333
Thoughts about ending one’s own life	0.422	0.535	0.481	0.701	0.578	0.857	0.735
Difficulties in making decisions	0.125	0.470	0.129	0.536	0.378	0.505	0.025^*^
Feeling nervous, tense, preoccupied	0.006^*^	0.076	0.321	0.299	0.158	0.232	0.062
Loss of satisfaction with DLAs	0.027^*^	0.041^*^	0.212	0.132	0.167	0.470	0.042^*^

CRAS = Centro de Referência de Assistência Social; DLAs = daily life activities.

* p < 0.05.

In terms of physical demands, standing for long periods was significantly associated with musculoskeletal pain (p = 0.026). Walking a lot was associated with easy tiredness (p = 0.023). Working without breaks was statistically associated with difficulty making decisions (p = 0.034). Sitting for long periods and working night shifts were significantly associated with a loss of satisfaction with DLAs (p = 0.004 and p = 0.017, respectively). Finally, rotating shifts were significantly associated with headaches (p = 0.045). The other work demands did not have significant associations with health perceptions (Table 4).

**Table 4:** Health aspects of CRAS social welfare workers associated with job physical demands (n = 41)

	Physical demands due to the organization of work (p-value)							
	Posture that leads to pain or discomfort	Standing	Sitting	Walking too much	Carrying or lifting weight	Working without break	Rotating shifts	Night shift
Musculoskeletal pain	0.360	0.026^*^	0.327	0.054	0.102	0.373	0.324	0.568
Sleeping badly	0.594	0.117	0.428	0.572	0.338	0.136	0.539	0.568
Poor digestion	0.213	0.257	0.435	0.257	0.569	0.319	0.592	0.637
No appetite	0.691	0.428	0.572	0.572	0.495	0.256	0.731	0.470
Trembling hands	0.213	0.153	0.153	0.023^*^	0.241	0.377	0.290	0.637
Tires easily	0.163	0.487	0.091	0.260	0.059	0.139	0.475	0.287
Headache	0.370	0.640	0.333	0.640	0.470	0.466	0.045^*^	0.225
Thoughts about ending their life	0.407	0.735	0.735	0.735	0.343	0.107	0.538	0.381
Difficulties in making decisions	0.300	0.155	0.123	0.620	0.226	0.034^*^	0.063	0.087
Feeling nervous, tense, preoccupied	0.210	0.136	0.191	0.329	0.177	0.138	0.618	0.538
Loss of satisfaction with DLAs	0.173	0.568	0.004^**^	0.432	0.087	0.212	0.578	0.017^*^

CRAS = Centro de Referência de Assistência Social; DLAs = daily life activities.

* p < 0.05.

Among the psychosocial aspects, feeling that one’s safety is threatened was statistically significant in relation to symptoms of easy tiredness (p = 0.048) and nervousness, tension, and preoccupation (p = 0.016). Threatening the safety of personal effects was associated with musculoskeletal pain (p = 0.006). Experiencing aggression from users was statistically associated with headache (p = 0.047) and difficulty making decisions (p = 0.047). Experiencing aggression or threats from relatives/companions of health service users was associated with musculoskeletal pain (p = 0.037), easy tiredness (p = 0.032), nervousness, preoccupation, and tension (p = 0.022), and loss of satisfaction with DLAs (p = 0.025). Witnessing episodes of aggression or threats between coworkers and supervisors was significantly associated with symptoms of poor sleep (p = 0.011) and headaches (p = 0.030) (Table 5).

**Table 5:** Health aspects of CRAS workers associated to psychosocial aspects caused by the organization of work (n = 41)

	Psychosocial aspects caused by the organization of work (p-value)										
	Little time to eat	No food	No dining room or canteen	No room to rest	No personal effects locker	Threats to safety	Threats to personal effects	Aggression or threats made by the user	Aggression or threats made by companions	Aggression or threats from colleagues to users	Aggression or threats between colleagues/ superiors
Musculoskeletal pain	0.552	0.656	0.218	0.526	0.053	0.102	0.006^*^	0.051	0.037^*^	0.479	0.281
Sleeping badly	0.209	0.582	0.572	0.659	0.415	0.225	0.446	0.596	0.588	0.276	0.011^*^
Poor digestion	0.317	0.257	0.644	0.805	0.399	0.499	0.472	0.619	0.513	0.096	0.172
No appetite	0.232	0.237	0.096	0.951	0.154	0.461	0.296	0.521	0.636	0.146	0.192
Trembling hands	0.683	0.743	0.644	0.805	0.399	0.205	0.528	0.381	0.513	0.498	0.607
Tires easily	0.291	0.323	0.634	0.610	0.632	0.048^*^	0.068	0.065	0.032^*^	0.057	0.167
Headache	0.299	0.689	0.395	0.780	0.601	0.093	0.135	0.047^*^	0.059	0.545	0.030^*^
Thoughts about ending their own life	0.670	0.662	0.857	0.927	0.205	0.693	0.577	0.630	0.498	0.786	0.723
Difficulties in making decisions	0.461	0.149	0.495	0.707	0.261	0.107	0.073	0.047^*^	0.452	0.668	0.346
Nervousness, tension, or preoccupation	0.476	0.204	0.232	0.512	0.475	0.016^*^	0.055	0.078	0.022^*^	0.115	0.053
Loss of satisfaction with DLAs	0.408	0.180	0.470	0.732	0.360	0.066	0.135	0.122	0.025^*^	0.178	0.300

CRAS = Centro de Referência de Assistência Social; DLAs = daily life activities.

* p < 0.05.

## DISCUSSION

The results have shown a prevalence of social welfare workers and psychologists, mainly women and young adults, among the CRAS staff. Psychologists and social welfare workers are the most common professionals responsible for implementing SUAS and are part of the minimum team required at CRAS.^9^ This female presence is related to the fact that these professions are predominantly occupied by women.^17^ This finding is consistent with national data indicating a higher participation of women in the Brazilian public service workforce.^17^

The mean age of CRAS workers is lower than that of public sector workers in Brazil, and the educational level of the sample is higher than that of the general Brazilian population.^18^ This discrepancy is due to Política Nacional de Assistência Social (PNAS) requirements, which mandate that the minimum CRAS team include both technical and higher education professionals. Despite their high level of formal education, the income of CRAS workers was lower than the average real income of the broader Brazilian workforce.^18^ The employment structure in public services includes outsourced workers, interns, temporary workers, and those who have passed public selection processes.^19^ This variation in employment types is present in CRAS and may explain the income levels of the workers evaluated in this study.

Self-perceived health status is an index that reflects individuals’ perceptions of their own health.^20^ While it includes biological factors such as diagnosed disease, it is also culturally influenced by beliefs and behaviors, including the presence of risk factors, disease prevention, and well-being.^21^ Most social welfare workers in the study rated their health as good, which is better than the national mean. National data show 66.1% of Brazilians rate their health as good or very good, while 33.9% rate their health as poor.^20^ According to Alcântara et al.,^21^ social welfare workers who are exposed to excessive physical stress are more likely to rate their health as bad or very bad, which is consistent with our findings. This underscores the impact of the work environment on health perceptions, exposing workers to higher risks of illness, absenteeism and sick leave.

The main health complaints reported by workers were feeling nervous, preoccupied or tense; getting tired easily; having difficulty making decisions; and losing satisfaction with DLAs. Precarious jobs can have a significant impact on workers’ health and well-being, especially when equipment is inadequate, resources are insufficient, and available space is insufficient or unsafe.^21^ The results show that workers face poor or inadequate conditions in their working environment, and that there is a link between these conditions and workers’ health. The main physical demands reported by workers were standing or sitting for long periods, working without breaks, and rotating shifts. Symptoms associated with these demands included musculoskeletal pain, fatigue, difficulty making decisions, and loss of satisfaction with DLAs. These job demands mirror health problems that have been studied in various worker populations.^22,23^

Studies of workers in different fields confirm these findings. Dantas et al.^7^ showed that standing work, uncomfortable postures, intense work rhythms, and rotating shifts are associated with complaints of physical and mental fatigue, stress, lack of concentration and attention, and impaired judgment. Job demands such as prolonged standing, carrying heavy loads, maneuvering equipment, and constant movement are associated with musculoskeletal disorders.^22^ Excessive work pace, excessive workload, insufficient time to complete tasks, and frequent interruptions are also associated with symptoms such as mental fatigue, drowsiness, forgetfulness, nervousness, sleeplessness, and psychological distress.^23^

Characteristics of a poor working environment, such as inadequate ventilation, temperature, lighting, and lack of material resources, were significantly associated with the mental health of the workers studied. This relationship between psychological and emotional symptoms and precarious working conditions has been confirmed by other studies.^6,22,23^ According to Maciel et al.,^6^ poor working conditions, such as unmaintained equipment, broken chairs and tables, poor lighting, and lack of equipment, lead to increased anxiety among workers. Other studies show that low material resources, noise, and an inadequate physical environment are associated with health problems such as anxiety, tension, nervousness, irritability, depression, sleep disorders, stress, and psychological disorders.^23^

Specific occupational health and safety interventions aimed at minimizing physical and mental harm are a potential strategy for promoting the health of these workers. These interventions should focus on improving the working environment by addressing issues such as inadequate temperature control, poor ventilation and lack of equipment, resources, materials, and furniture. The implementation of these measures is expected to primarily prevent physical harm, mental exhaustion and sick leave among social service workers.

Most CRAS in Brazil are in rented buildings, which may explain the inadequate physical space.^24^ These rented buildings are often unsafe, improvised, or unhealthy, resulting in poor working environments. National data show most CRAS do not comply with safety and accessibility guidelines and have environmental barriers that affect both the work of staff and the use of social services.^10,21^ Castro^24^ suggests that new CRAS units should be built with appropriate surroundings. An adequate physical space provides better working conditions and more efficient service to the population. The analysis of the CRAS environments highlights the importance of the context in which social assistance services are provided and the health indicators of workers, thereby enhancing intersectoral action. Long-term management and governance policies aimed at the adequacy and accessibility of public buildings can generate benefits in several areas, including health and social assistance. Therefore, public management strategies should focus on urban improvement, creating a favorable, healthy and sustainable environment with fewer barriers. This approach would allow for greater mobility, autonomy, safety, and access to quality public services.

Insecurity and episodes of aggression were present in CRAS. The characteristics of CRAS may contribute to negative perceptions of job security. Experiencing aggression or threats or witnessing episodes of aggression and threats among coworkers and supervisors were significantly associated with several health problems. In Brazil, 3.1% of the population aged 18 years or older have been victims of violence or aggression by strangers, and 18.4% of these incidents occurred at work.^25^ The proportion of aggression or violence by known persons was 2.5%, with 11.9% of these incidents occurring at work.^25^ Recent research have shown an association between episodes of violence and health problems among workers.^8,11^ Mental aggression, lack of communication, lack of collective defenses, and psychological stress are associated with chronic headaches, fatigue, exhaustion, and irritability.^8,11^

Workplace violence negatively affects the health and safety of workers and is an important occupational health issue. Aytaç & Dursun^26^ show a relationship between workplace aggression and symptoms of depression and stress, which negatively affect job satisfaction. Health and social service workers are more likely to be exposed to violent situations, especially when they work directly with people who have a history of drug or alcohol abuse, when they work alone, when the workplace architecture is in poor condition, when there are no means of communicating an emergency, and when they work in neighborhoods with high crime rates.^27^ This is the reality for workers at CRAS in Brazil.

Violence prevention programs in social services should include training to recognize violent behavior and de-escalation techniques as well as structural and administrative measures to control violence (e.g., alarms, surveillance, and increased staffing). In addition, measures to reduce occupational stress, such as wellness courses and organizational improvements, are essential.^8^ Organizational support and workplace violence training are also cited as effective strategies to help workers cope with workplace violence.^28^ Team-based training can improve the interpersonal skills of workplace violence victims and increase perceived support from coworkers, both of which can prevent workplace violence incidents and their recurrence.^28^

This research has some limitations that should be addressed. One limitation is that we could not analyze health complaints in relation to gender and professional role. Our sample consisted mainly of women, psychologists and social workers. As a result, the generalization of these data is consistent with the reality of social service teams and the number of professionals working in CRAS in Brazil. Despite the small sample size, almost all CRAS workers were evaluated, and the profile of the sample is similar to that of CRAS workers described in other studies.^17,29^

The cross-sectional nature of the study does not allow us to identify the cause of the health complaints. However, this methodological design was useful for analyzing our hypothesis of an association between these symptoms and working conditions. The health characteristics assessed in this study are dynamic, complex, and influenced by various factors throughout life. Longitudinal studies are therefore needed to examine the impact of working environment conditions on the health of this group of workers on an ongoing basis. Such information may have implications for redesigning the practice of occupational health services. For future studies, we also suggest analyzing changes in the health status of workers over time, especially after the COVID-19 pandemic. The current post-pandemic scenario has increased the number of at-risk and vulnerable people, which has further increased the demands on CRAS workers. However, this new reality has not been translated into investments and actions aimed at improving the working conditions of this occupational group.

Currently, researchers use workers’ self-perceptions of various aspects of their work and relate their health self-perceptions to their exposure to occupational risk factors and working conditions. Therefore, this study is relevant and advances the use of measures that incorporate health self-perceived as a subjective, individual measure related to worker health.

## CONCLUSIONS

This study found that CRAS workers were predominantly female, highly educated, and had healthy lifestyles. However, there were high rates of negative health perceptions and chronic pain among the workers. Poor working conditions, high physical demands, and psychosocial factors related to violence and feelings of insecurity were common. The results confirmed the relationship between poor working conditions, lack of safety, episodes of violence and health problems.

Few studies have focused on social service workers as a target population, which underscores the importance of analyzing their health problems due to the occupational hazards that affect this group. Therefore, it is crucial to develop plans to reduce risk factors in CRAS work environments, such as interventions that provide a more comfortable environment through ergonomic analysis to minimize physical demands. In particular, further and more in-depth research is needed on the presence of violence in CRAS workplaces as well as the planning and development of preventive measures aimed at this psychosocial aspect.
